# Waxes from Long-Chain Aliphatic Difunctional Monomers

**DOI:** 10.1021/acssuschemeng.3c06951

**Published:** 2023-11-14

**Authors:** Marcel Eck, Celia Stoltze, Stefan Mecking

**Affiliations:** Department of Chemistry, University of Konstanz, Universitätsstr. 10, 78457 Konstanz, Germany

**Keywords:** Wax, polyethylene-like, biodegradable, biobased, long-chain polyester

## Abstract

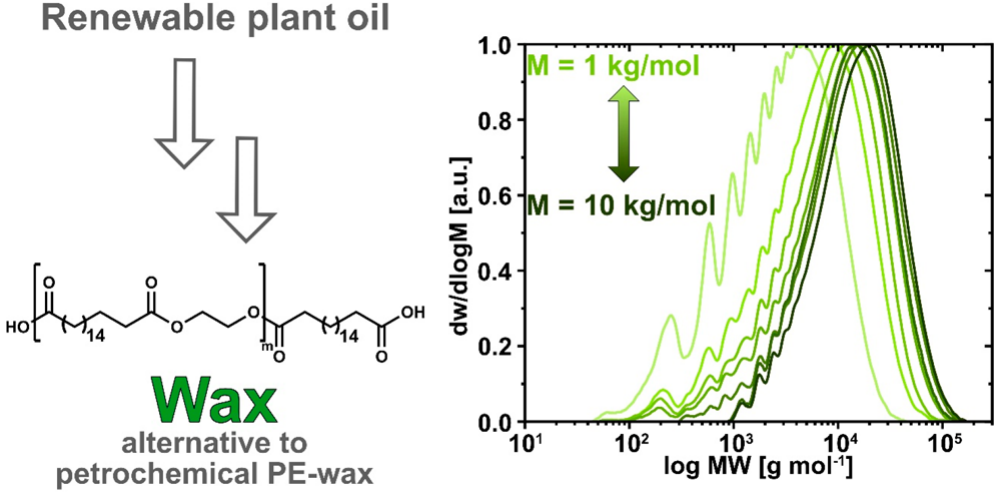

Petrochemical polyethylene waxes (*M*_n_ = 800–8000 g/mol for commercial Ziegler waxes) as additives, lubricants, and release agents are essential to numerous products and production processes. The biodegradability of this class of compounds when unintentionally released to the environment is molar mass dependent and subject to ongoing discussions, and alternatives to conventional polyethylene waxes are desirable. By employing bottom-up and top-down approaches, that is nonstoichiometric A_2_ + B_2_ polycondensation and chain scission, respectively, linear waxes with multiple in-chain ester groups as biodegradation break points could be obtained. Specifically, waxes with 12,12 (WLE-12,12, WLE = waxes linear ester) and 2,18 (WLE-2,18) carbon atom linear ester repeat unit motifs were accessible over a wide range of molar masses (*M*_n_ ≈ 600–10 000 g/mol). In addition to the molar mass, the type of end group functionality (i.e., methyl ester, hydroxy, or carboxylic acid end groups) significantly impacts the thermal properties of the waxes, with higher melting points observed for carboxylic acid end groups (e.g., *T*_m_ = 83 °C for carboxylic acid-terminated WLE-12,12 with *M*_n,NMR_ = 1900 g/mol, *T*_m_ = 92 °C for WLE-2,18 with *M*_n,NMR_ = 2200 g/mol). A HDPE-like orthorhombic crystalline structure and rheological properties comparable to a commercial polyethylene wax suggest WLE-12,12 and WLE-2,18 are viable biodegradable and biosourced alternatives to conventional, petrochemical polyethylene waxes.

## Abstract 2

Renewable and biodegradable alternatives to polyethylene waxes based on difunctional long-chain aliphatic monomers.

## Introduction

Polyolefin waxes, produced on a scale of 100 000 tons per year (600 000 tons/year in 2014), are employed as auxiliaries in numerous products and production processes.^1^ Ethylene-based low- and high-density polyethylene waxes represent the largest group of polyolefin waxes. They can be produced via high pressure free radical chain growth and Ziegler or metallocene catalysis, respectively.^2^ Their crystallinity, hydrophobicity, and low melt viscosities (due to relatively low molar masses of *M*_n_ = 800–8000 g/mol for commercial Ziegler waxes) predestine both types of polyethylene waxes for applications as additives in paints and coatings as well as in plastics processing as lubricants and release agents. The degradability of this class of compounds when unintentionally released to the environment, however, varies strongly with the molar mass of the waxes and is subject to ongoing discussions.^3−6^ To prevent accumulation and concomitant adverse ecological effects,^7^ entirely nonpersistent alternatives to polyethylene waxes are desired.^8^

Natural carnauba and montan waxes are mixtures of compounds mainly consisting of monoesters of long-chain acids and alcohols and free long-chain acids (C_16_–C_34_).^1,9^ The areas of application of these waxes extracted from palm leaves and lignite,^10^ respectively, and of polyolefin waxes overlap. However, due to a fluctuating supply and an inconsistent quality, such natural waxes cannot be considered a viable replacement for polyolefin waxes.

Low densities of ester or carbonate groups incorporated into a polyethylene chain can act as break points and facilitate closed-loop chemical recycling while retaining the crystalline structure and outstanding mechanical properties of HDPE.^11^ A prominent example for a recyclable, HDPE-like material is polyester-18,18 (PE-18,18, *T*_m_ = 99 °C), which can be obtained by polycondensation of commercially available, plant oil-based 1,18-octadecanedioic acid (C_18_-diacid) and its corresponding C_18_-diol.^12^ We recently found that the substitution of the C_18_-diol by a short-chain congener, that is ethylene glycol (C_2_), additionally endows the resulting polyester-2,18 (PE-2,18) with biodegradability as demonstrated by respirometric tests.^13^ The material’s desirably high melting point (*T*_m_ = 96 °C) and HDPE-like material properties are retained at the same time.

Waxes and polymers, in general, possess very different physical properties and application profiles. Notwithstanding, the polyethylene-like solid-state structure of the aforementioned polyesters encouraged an exploration of potential wax materials based on their monomer building blocks.

We now report alternatives to polyethylene waxes with multiple in-chain ester groups in their linear chains and their generation from biobased or fossil feedstocks.

## Experimental Section

### Materials

All chemicals were used as received without further purification. 1,18-Octadecanedioic acid was purchased from Elevance Renewable Sciences Inc. PE-2,18 was prepared by a reported procedure.^13^ 1,12-Dodecanedioic acid (99%), 1,12-dodecanediol (99%), and dibutyltin oxide (DBTO, for synthesis) were purchased from Sigma-Aldrich. Dimethyl 1,12-dodecanedioate (>98%) was purchased from TCI. High-density polyethylene Purell GB 7250 from LyondellBasell and micronized polyethylene wax E 0915 M from Deurex were used as reference materials. Deuterated solvents for NMR spectroscopy were purchased from Eurisotop. All manipulations involving air- and/or moisture-sensitive substances were carried out under inert atmosphere using standard Schlenk and glovebox techniques.

### Small-Scale Oligomerization Experiments for WLE-12,12 Waxes with Carboxylic Acid End Groups

Ten milliliter glass inlets were charged with 1,12-dodecanedioic acid (1.00 g, 4.3 mmol, 1.0 equiv), DBTO (3.2 mg, 0.3 mol %), a stir bar, and variable amounts of 1,12-dodecanediol to achieve different degrees of oligomerization (cf. Table S3 in the Supporting Information). The 10 charged inlets were placed in a heating block within a 1 L steel vessel; the vessel was sealed and repeatedly evacuated and purged with nitrogen (4×). The temperature was raised to 150 °C (temperature of the heating block surrounding the 1 L vessel), and the mixtures were stirred at 300 rpm. After 1 h at ambient pressure, the pressure was reduced to 100 mbar over the course of 1 h using a membrane pump. Stirring was continued overnight, and the vessel was allowed to cool to room temperature. The products were obtained as colorless solids. The waxes were characterized as obtained from the reactions without further workup.

### Small-Scale Oligomerization Experiments for WLE-12,12 Waxes with Methyl Ester End Groups

Ten milliliter glass inlets were charged with dimethyl 1,12-dodecanedioate (1.00 g, 3.9 mmol, 1.0 equiv), DBTO (3.0 mg, 0.3 mol %), a stir bar, and variable amounts of 1,12-dodecanediol to achieve different degrees of oligomerization (cf. Table S4). The 10 charged inlets were placed in a heating block within a 1 L steel vessel, and the reactions were conducted as outlined for WLE-12,12 waxes with carboxylic acid end groups. During oligomerization, a vacuum of 300 mbar instead of 100 mbar was applied on account of the higher volatility of the condensation product methanol in comparison to water.

### Small-Scale Oligomerization Experiments for WLE-12,12 Waxes with Hydroxy End Groups

Ten milliliter glass inlets were charged with 1,12-dodecanediol (1.00 g, 4.9 mmol, 1.0 equiv), DBTO (3.7 mg, 0.3 mol %), a stir bar, and variable amounts of dimethyl 1,12-dodecanedioate to achieve different degrees of oligomerization (cf. Table S5). The 10 charged inlets were placed in a heating block within a 1 L steel vessel, and the reactions were conducted as outlined for the oligomerizations for WLE-12,12 waxes with methyl ester end groups.

### Larger-Scale Oligomerization Experiment for WLE-12,12 Wax (Target *M*_n_ ≈ 2.4 kg/mol) with Carboxylic Acid End Groups

A 1 L round-bottom flask was charged with 1,12-dodecanedioic acid (100.0 g, 434.2 mmol, 1.0 equiv), 1,12-dodecanediol (74.7 g, 369.1 mmol, 0.85 equiv), DBTO (324.3 mg, 0.3 mol %), and an elliptical PTFE-coated stir bar with a rare earth core. The flask was placed in a heating block, and a condenser flask was connected to collect the volatiles released during the polymerization reaction. The setup was repeatedly evacuated and purged with nitrogen (4×), and the temperature was raised to 150 °C while being stirred at 300 rpm. After 1 h of reaction time at ambient pressure, the pressure was gradually reduced to 100 mbar over the course of 1 h. After 18 h of oligomerization at a pressure of 100 mbar, the hot melt was precipitated in −30 °C 2-propanol. The solvent was filtered off, and the product was dried in a vacuum drying oven at 50 °C affording colorless WLE-12,12 (145.0 g).

### Small-Scale Syntheses of WLE-2,18 Waxes via Chain Scission

Eight milliliter glass vials were charged with finely ground polymeric PE-2,18 (1.00 g, *M*_n,SEC_ = 48 kg/mol vs PS standards) and variable amounts of 1,18-octadecanedioic acid to achieve different degrees of oligomerization (cf. Table S6). The solids were mixed, a stir bar was added, and the sealed vials were placed in a heating block. The temperature was increased to 180 °C, and the reaction mixtures were stirred for 5 h at 100 rpm. Subsequently, the temperature was reduced to 150 °C, and the reactions were stirred for a further 12 h. Finally, the waxes were allowed to cool to room temperature and were characterized without further workup.

### Larger-Scale Synthesis of WLE-2,18 Wax (Target *M*_n_ = 2 kg/mol) via Chain Scission

A 250 mL round-bottom flask equipped with an elliptical PTFE-coated stir bar with a rare earth core was charged with PE-2,18 (40.5 g, *M*_n,SEC_ = 48 kg/mol vs PS standards) and 1,18-octadecanedioic acid (7.6 g, 24.2 mmol). The setup was repeatedly evacuated and purged with nitrogen (4×) and then loosely sealed with a stopper to facilitate pressure compensation, if necessary. The round-bottom flask was placed in a heating block, the temperature was raised to 180 °C, and the mixture was stirred at 100 rpm for 24 h. Subsequently, the temperature was reduced to 120 °C, and the stirring rate was increased to 500 rpm. These conditions were maintained for a further 40 h. Finally, the hot melt was precipitated in −30 °C 2-propanol. The solvent was filtered off, and the solid was washed with acetone. The obtained off-white product was dried in a vacuum drying oven at 50 °C, affording WLE-2,18 (41.2 g).

### Characterization and Processing

Nuclear magnetic resonance (NMR) spectra were recorded on a Bruker Avance III 400 spectrometer. Chemical shifts were referenced to the resonance of the solvent (residual proton resonances for ^1^H spectra). Mestrenova software by Mestrelab Research S.L. (version 14.1.2) was used for data evaluation.

Molar masses were determined by size-exclusion chromatography (SEC) in chloroform at 35 °C on a PSS SECcurity^2^ instrument, equipped with PSS SDV linear M columns (2 × 30 cm, additional guard column) and a refractive index detector (PSS SECcurity^2^ RI). A standard flow rate of 1 mL min^–1^ was used. Molar masses were determined versus low dispersity polystyrene (PS) standards (software: PSS WinGPC, version 8.32).

Differential scanning calorimetry (DSC) was carried out on a Netzsch DSC 204 F1 instrument (software: Netzsch Proteus Thermal Analysis, version 6.1.0) with a heating/cooling rate of 10 K min^–1^. Data reported are from second heating cycles.

Wide angle X-ray scattering (WAXS) diffractograms were recorded on a D8 Discover instrument (Bruker) with a Vantec detector on disc-shaped specimens (ca. 5.5 cm diameter) obtained by allowing a wax melt (*T* = 200 °C) to cool to room temperature in an aluminum pan. Crystallinity of waxes (χ_WAXS_) was determined from the WAXS patterns as χ_WAXS_ = [*A*_c_(110) + *A*_c_(200)]/[*A*_c_(110) + *A*_c_(200) + *A*_a_] where *A*_c_ refers to the integrated area of the Bragg reflections from the orthorhombic crystal and *A*_a_ to the integrated area of the amorphous halo. A Voigt fit was used.

Surface free energies were determined on aforementioned disc-shaped specimens by the method of Fowkes on a drop shape analyzer DSA25 by KRÜSS.^14^

Rheological frequency sweep experiments were performed on an ARES-G2 rheometer over a range of 1 to 500 rad s^–1^ with cone–plate geometry (diameter: 50 mm) at 140 °C.

Thermogravimetric analysis was performed on a Netzsch STA 449 F3 Jupiter. Measurements were performed with a 250 mL/min flow rate of a synthetic 80:20 mixture of N_2_/O_2_ at a heating rate of 10 K/min from 30 to 1000 °C.

## Results and Discussion

### Waxes with 12,12 Carbon Atom Linear Ester Repeat Units (WLE-12,12)

**Figure 1:**
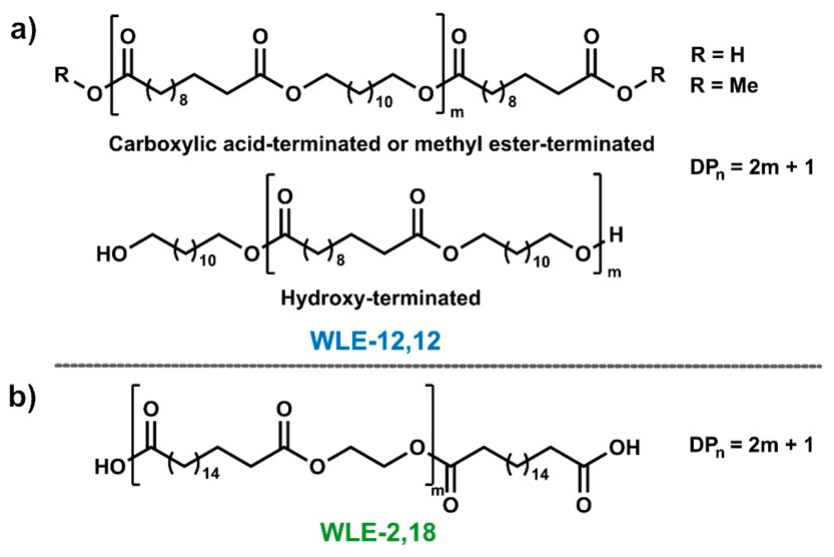
Chemical structures of waxes. (a) WLE-12,12 waxes with carboxylic acid (R = H) and methyl ester (R = Me) end groups (top) and WLE-12,12 wax with hydroxy end groups (bottom). (b) WLE-2,18 wax obtained by chain scission with C_18_-diacid.

WLE-12,12 waxes were synthesized via dibutyltin oxide-catalyzed condensation of dimethyl 1,12-dodecanedioate^15^ (C_12_-dimethyl ester) and 1,12-dodecanediol (C_12_-diol). The degrees of polymerization DP_*n*_ of the waxes were adjusted within a range from 3 to 12 (i.e., target *M*_*n*_ ≈ 600–2400 g/mol) by adjusting the ratio *r* of the two reacting monomers (cf. eq S2 in the Supporting Information).^16^ By employing either the C_12_-dimethyl ester or the C_12_-diol in excess, waxes with primarily methyl ester and hydroxy end groups, respectively, could be obtained (Figure 1a). The synthesis of WLE-12,12 waxes with carboxylic acid end groups was achieved analogously by reacting excess 1,12-dodecanedioic acid (C_12_-diacid) with C_12_-diol.

**Figure 2:**
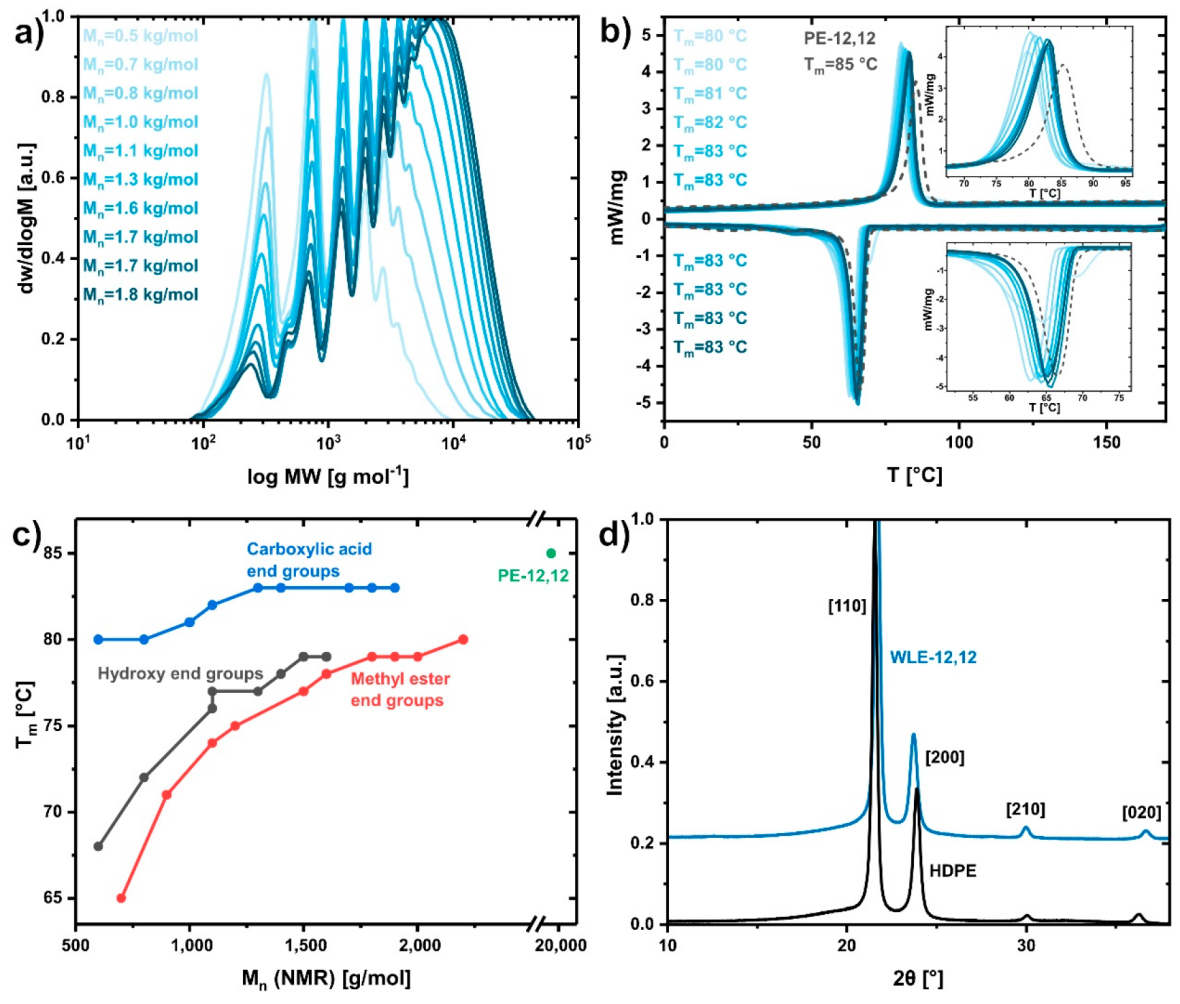
Characterization data for WLE-12,12 waxes, obtained by nonstoichiometric condensation. (a) SEC traces and number-average molar masses *M*_n_ (vs PS standards) of carboxylic acid-terminated WLE-12,12 waxes with target DP_*n*_ values of 3–12. (b) DSC traces and peak melting points *T*_m_ of carboxylic acid-terminated WLE-12,12 waxes with target DP_*n*_ values of 3–12. The DSC trace of polymeric PE-12,12 is shown for comparison (dashed line). (c) Relationship between the molar mass determined via ^1^H NMR end group analysis and the peak melting point for methyl ester-, hydroxy-, and carboxylic acid-terminated WLE-12,12 waxes. (d) WAXS diffractogram of carboxylic acid-terminated WLE-12,12 wax (*M*_n,NMR_ = 2300 g/mol) in comparison to HDPE. Traces are shifted vertically for clarity.

Size exclusion chromatography (SEC) and ^1^H NMR end group analysis confirmed the control over the molar mass and end group functionality of the waxes via monomer stoichiometry (Figure 2a, cf. Figures S1–S10 for additional SEC and ^1^H NMR data of WLE-12,12 waxes). Note that molar masses determined by SEC (vs polystyrene) are dependent on the hydrodynamic behavior of the oligomers and therefore are instructive only for relative comparison of waxes with the same type of end group (cf. Figures S2, S4, and S8 for comparison of molar masses determined by SEC and ^1^H NMR end group analysis for waxes with different end group functionalities). Absolute number-average molar masses determined by ^1^H NMR end group analysis are better suited for comparison of different types of waxes (cf. Supporting Information for molar mass determination via end group analysis of ^1^H NMR spectra).

Due to the oligomeric character of the waxes, an influence of the molar mass and type of end group on the thermal properties can be expected. Differential scanning calorimetry (DSC) revealed broad thermal transitions with multiple peaks observed for methyl ester- and hydroxy-terminated waxes (cf. Figures S5 and S9 for additional DSC data). The melting and crystallization peaks were strongly influenced by an increasing molar mass and shifted to higher temperatures (e.g., peak *T*_m_ of 65 °C and 80 °C for methyl ester-terminated WLE-12,12 with *M*_n,NMR_ of 700 g/mol and 2200 g/mol, respectively; Figure 2c). In contrast, carboxylic acid-terminated waxes exhibited single and narrow melting and crystallization peaks comparable to those of polymeric PE-12,12 (Figure 2b). The melting and crystallization points of these waxes (*T*_m_ increasing only from 80 °C to 83 °C) proved to be less dependent on the molar mass of the waxes and approached the melting point of polymeric PE-12,12 (*T*_m_ = 85 °C, Figure 2c). We assume that the conspicuously different thermal properties of the carboxylic acid-terminated waxes are related to the end groups’ capability to form intermolecular hydrogen bonds, facilitating a favorable arrangement of neighboring oligomer chains. Comparison of the melting and crystallization enthalpies indicated a higher crystallinity of the waxes in comparison to reference polymer (e.g., Δ*H*_m_ = 149 J/g for carboxylic acid-terminated WLE-12,12 wax with *M*_n,NMR_ = 1900 g/mol vs Δ*H*_m_ = 134 J/g for polymeric PE-12,12; cf. Tables S7–S9 for additional data on thermal properties).

Due to the advantageous thermal properties in comparison to methyl ester- and hydroxy-terminated waxes, a WLE-12,12 wax with carboxylic acid end groups (*M*_n,NMR_ = 2300 g/mol) was synthesized on a larger 145 g scale for further investigation of its solid-state properties (cf. Figures S11–S16 for additional characterization data and photographs of the experimental setup and product). Characterization of the brittle, colorless material via WAXS revealed an orthorhombic crystalline structure akin to HDPE and a crystallinity of χ = 75% (Figure 2d, χ ≈ 71% for polymeric PE-12,12). The presence of in-chain ester groups and the high density of carboxylic acid end groups are reflected in a decreased water contact angle and an increased surface free energy (SFE) of the WLE-12,12 wax compared to HDPE (82° vs. 97° and SFE = 42 mN m^–1^ vs. 32 mN m^–1^, cf. Table S1).^17^

### Waxes with 2,18 Carbon Atom Linear Ester Repeat Units (WLE-2,18)

The volatility of the ethylene glycol monomer under oligomerization conditions complicates the adjustment of the molar mass of WLE-2,18 waxes by the method employed for the WLE-12,12 waxes, that is, via the stoichiometry of the reacting monomers (Figure 1b). Hence, a top-down approach by chain scission^18^ was pursued to obtain the desired waxes. High molecular weight PE-2,18 was reacted with C_18_-diacid at elevated temperatures in a closed vessel such that no volatiles (formed water) are removed. The final molar mass was adjusted by the amount of C_18_-diacid agent employed (cf. eq S3). Note that top-down processes are used on an industrial scale to produce polyethylene waxes, in this case by uncontrolled thermal degradation of higher molar mass polyethylene.^1^

**Figure 3:**
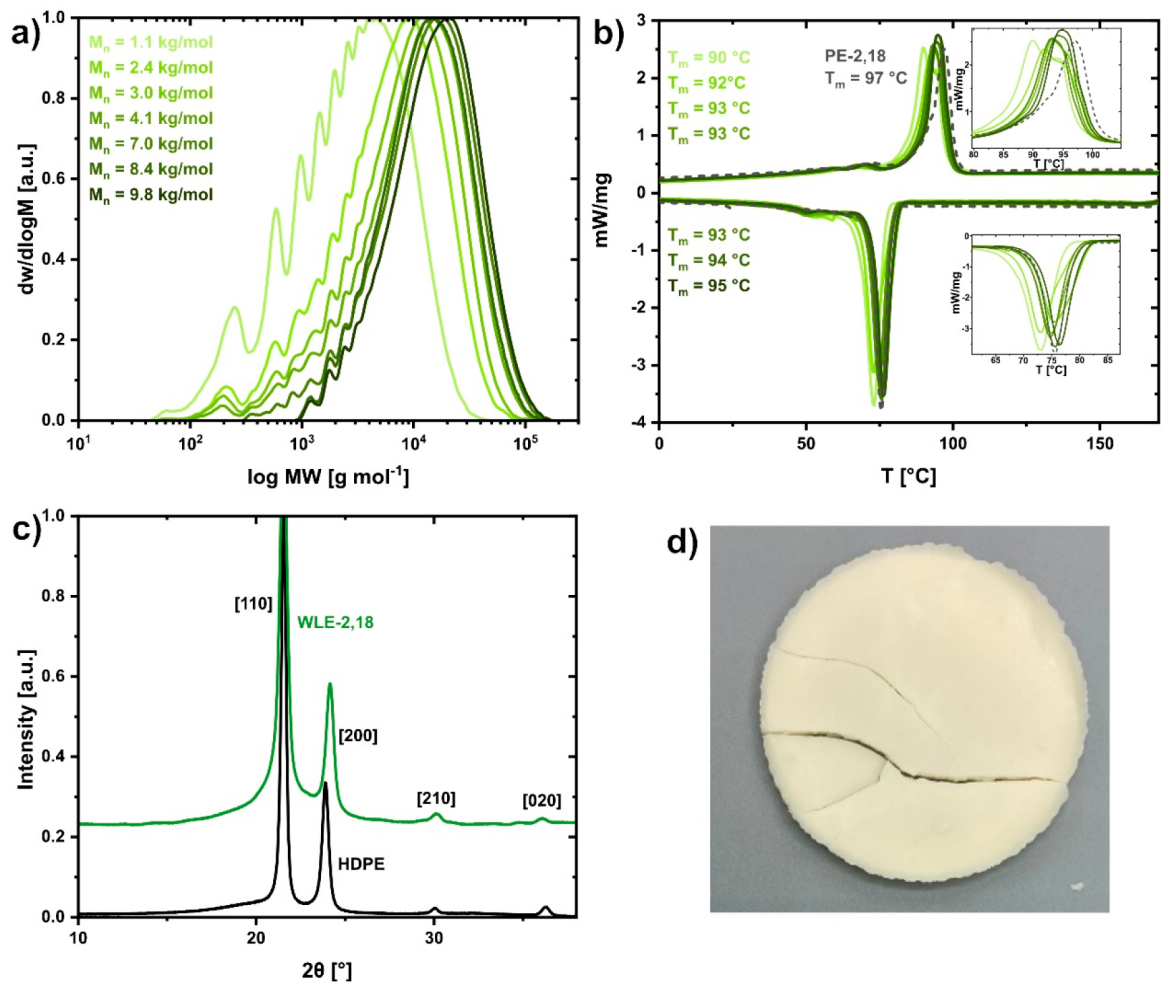
Characterization data for WLE-2,18 waxes obtained by chain scission. (a) SEC traces and number-average molar masses *M*_n_ (vs PS standards) of WLE-2,18 waxes with target molar masses *M*_n_ of 1, 2, 3, 4, 6, 8, and 10 kg/mol. (b) DSC traces and peak melting points *T*_m_ of WLE-2,18 waxes with target molar masses of 1, 2, 3, 4, 6, 8, and 10 kg/mol in comparison to polymeric PE-2,18 (dashed line). (c) WAXS diffractogram of WLE-2,18 wax (*M*_n,SEC_ = 1600 g/mol) in comparison to HDPE. Traces are shifted vertically for clarity. (d) Photograph of a disc-shaped specimen for WAXS and tensiometry measurements illustrating the brittle nature and off-white color of WLE-2,18 wax.

Chain scission facilitated the synthesis of WLE-2,18 waxes over a wide molar mass range of *M*_*n*_ ≈ 1000–10 000 g/mol as demonstrated by SEC measurements (Figure 3a; determination of molar masses from ^1^H NMR spectra was complicated for WLE-2,18 due to overlap of end group resonances, cf. Figures S17 and S19). Note that alternative chain scission by glycolysis with ethylene glycol of PE-2,18 was also found viable. The thermal properties of the WLE-2,18 waxes with *M*_*n*,SEC_ ≥ 4000 g/mol are comparable to the carboxylic acid-terminated WLE-12,12 waxes exhibiting single and narrow thermal transitions with melting points close to the melting point of polymeric PE-2,18 (Figure 3b). For WLE-2,18 waxes with lower molar masses, two overlapping melting transitions were observed. In agreement with the WLE-12,12 waxes, the melting and crystallization enthalpies indicated a crystallinity higher than for the reference polymer (e.g., Δ*H*_m_ = 135 J/g for WLE-2,18 wax with *M*_*n*,SEC_ = 2400 g/mol vs Δ*H*_m_ = 115 J/g for polymeric PE-2,18; cf. Table S10 for additional data on thermal properties).^13^

For further investigation of the solid-state properties, also a WLE-2,18 wax (*M*_*n*,SEC_ = 1600 g/mol) was synthesized on a larger 40 g scale (cf. Figures S19–S23 for additional characterization data and photograph of experimental setup). Akin to the WLE-12,12 wax, WAXS revealed a HDPE-like orthorhombic solid-state structure and a similar crystallinity of χ ≈ 72% (Figure 3c, χ ≈ 66% for polymeric PE-2,18).^13^ The surface of the brittle, off-white material exhibited a water contact angle and a surface free energy comparable to the carboxylic acid-terminated WLE-12,12 wax (81° and SFE = 43 mN m^–1^; Figure 3d, cf. Table S2). The molten material’s low complex viscosity (ca. 0.1 Pa·s at 140 °C) is in the typical range of waxes^1^ and compares to a commercial PE wax (ca. 0.01 Pa·s at 140 °C for a reference material of somewhat lower molar mass of *M*_*n*,NMR_ ≈ 700 g/mol, *T*_m_ = 98 °C, cf. Figure S24 for DSC trace).

A major area of application for PE waxes is additives in paints and coatings that enhance their scratch and abrasion resistance and control glossiness, transmission and haze, and surface slip. To this end, in various coating applications micronized WLE-2,18 performs on par with a commercial HDPE wax.

## Conclusions

Waxes with linear hydrocarbon chains comprising a low density of multiple in-chain ester groups (8 mol % ester vs. methylene for 12,12 repeat units; 10 mol % for 2,18 repeat units) and HDPE-like crystalline structures can be obtained over a wide range of molar masses (*M*_n_ ≈ 600–10 000 g/mol) via bottom-up nonstoichiometric oligomerization or top-down chain scission. This covers the molar mass range of polyethylene waxes as well as Fischer–Tropsch waxes.^1^ At the same time, accumulation of waxes released from applications into the environment appears much less problematic due to the proven biodegradability of similar higher molar mass polyesters.^13^ The waxes, which as a component in various coating systems perform on par with a commercial HDPE wax, can be sourced from biobased renewable as well as fossil-based diacid and diol starting materials.

## Supporting Information Available

Supplementary methods and data, including additional characterization of waxes (NMR spectra, SEC chromatograms, DSC traces, TGA traces, surface tension data), target molar mass of WLE-12,12 waxes via nonstoichiometric condensation, target molar mass of WLE-2,18 waxes via chain scission, molar mass determination via ^1^H NMR end group analysis, additional characterization of reference polyethylene wax (DSC trace); supplementary tables, including overview of monomer stoichiometries employed in the syntheses of WLE-12,12 waxes, overview of amounts of C_18_-diacid employed in the syntheses of WLE-2,18 waxes via chain scission, overview of thermal and molar mass properties of WLE-12,12 waxes, overview of thermal and molar mass properties of WLE-2,18 waxes (PDF)

## Abbreviations

WLE-12,12: wax with 12,12 carbon atom linear ester repeat units
WLE-2,18: wax with 2,18 carbon atom linear ester repeat units
PE-12,12: polyester-12,12
PE-2,18: polyester-2,18
PE-18,18: polyester-18,18
HDPE: high-density polyethylene
