# Supplementary material for: Waxes from Long-Chain Aliphatic Difunctional Monomers
Source: ACS Sustain Chem Eng. 2023 Nov 14;11(47):16716–21. doi: 10.1021/acssuschemeng.3c06951 (PMC10685719; doi:10.1021/acssuschemeng.3c06951)
Supplement: Supplementary file 1 — sc3c06951_si_001.pdf [file sc3c06951_si_001.pdf]

Supplementary Information

Waxes from long-chain aliphatic difunctional monomers

Marcel Eck, Celia Stoltze, and Stefan Mecking*

Chair of Chemical Materials Science, University of Konstanz,
Department of Chemistry, Universitätsstraße 10, 78457 Konstanz, Germany.

* stefan.mecking@uni-konstanz.de, Fax: +49 7531 885152
Tel.: +49 7531 882593

Number of pages: 24

Number of figures: 24

Number of tables: 10

Table of Contents

S1. Supplementary Methods and Data 3

Target molar mass of WLE-12,12 waxes *via* monomer stoichiometry 3

Target molar mass of WLE-2,18 waxes *via* chain scission with C_{18}-diacid 3

Additional characterization data for WLE-12,12 waxes with carboxylic acid end groups 4

Additional characterization data for WLE-12,12 waxes with methyl ester end groups 5

Additional characterization data for WLE-12,12 waxes with hydroxy end groups 7

Additional characterization data for WLE-12,12 wax synthesized on a larger scale 9

Additional characterization data for WLE-2,18 waxes synthesized *via* chain scission 12

Additional characterization data for WLE-2,18 wax synthesized on a larger scale 13

Determination of molar masses M_n *via* ^{1}H NMR end group analysis 16

Additional characterization data for commercial PE wax 17

S2. Supplementary Tables 18

Tabular overview of monomer stoichiometries employed in the syntheses of WLE-12,12 waxes 18

Tabular overview of amount of C_{18}-diacid employed in the syntheses of WLE-2,18 waxes *via* chain scission 19

Tabular overview of thermal and molar mass properties of WLE-12,12 waxes 20

Tabular overview of thermal and molar mass properties of WLE-2,18 waxes 23

References 24

S1. Supplementary Methods and Data

Target molar mass of WLE-12,12 waxes *via* monomer stoichiometry

The degrees of polymerization DP_n of the WLE-12,12 waxes were adjusted employing Carother's equation for non-stoichiometric A_2 + B_2 polycondensation reactions at complete conversion (**Equation S 1**, $r = N_{A2}/N_{B2}$).[1]

Equation S 1. $$DP_n = \frac{1+r}{1-r}$$

Rearrangement of **Equation S 1** yields **Equation S 2** which gives the ratio of the two reacting monomers A_2 and B_2, r, required to obtain oligomers with a desired DP_n (cf. **Table S 3**, **Table S 4**, **Table S 5**).

Equation S 2. $$r = \frac{DP_n-1}{DP_n+1}$$

Target molar mass of WLE-2,18 waxes *via* chain scission with C_{18}-diacid

The amount of C_{18}-diacid added as chain scission agent to obtain WLE-2,18 waxes of a desired molar mass from PE-2,18 was calculated according to **Equation S 3** (m_{SA} = mass of chain scission agent, $m_{Polymer}$ = mass of PE-2,18, M_{SA} = molar mass of chain scission agent, $M_{Oligomer}$ = molar mass of produced wax) (cf. **Table S 6**).[2]

Equation S 3. $$m_{SA} = \frac{m_{Polymer} \cdot M_{SA}}{M_{Oligomer}-M_{SA}}$$

Additional characterization data for WLE-12,12 waxes with carboxylic acid end groups

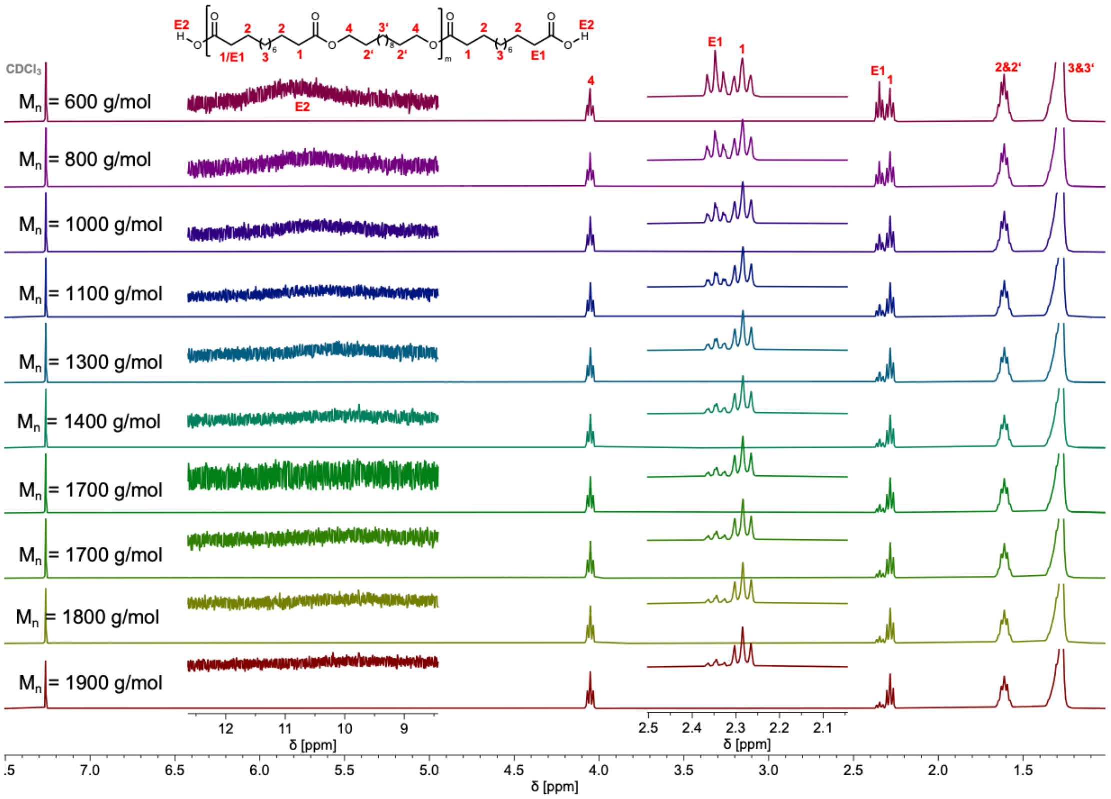

Figure S 1. Stacked ^{1}H NMR spectra (400 MHz, $CDCl_3$, 298 K) of WLE-12,12 waxes with carboxylic acid end groups. Number-average molar masses M_n were determined *via* end group analysis according to **Equation S 5**.

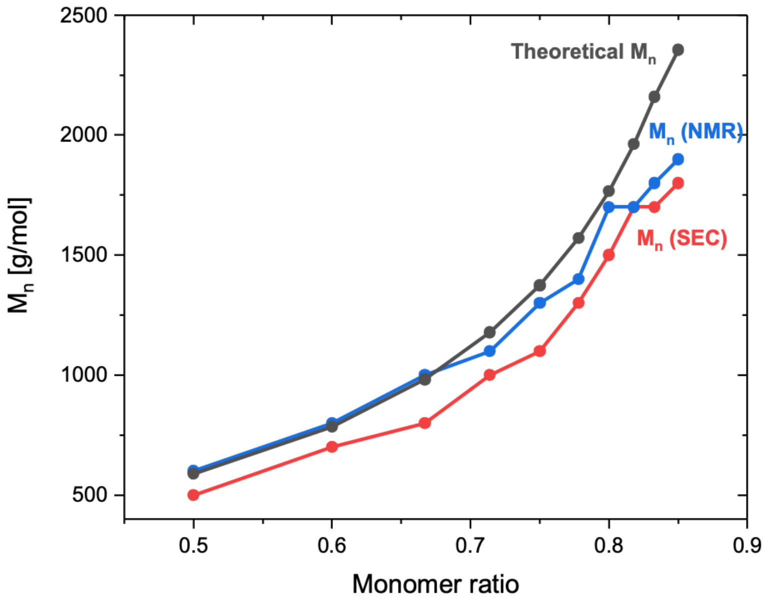

Figure S 2. Relationship between the ratio of C_{12}-diol and C_{12}-diacid employed in the oligomerization reactions and the theoretically expected molar mass M_n (grey), the molar mass M_n determined by ^{1}H NMR end group analysis (blue), and the molar mass M_n determined by SEC *vs.* PS standards (red).

Additional characterization data for WLE-12,12 waxes with methyl ester end groups

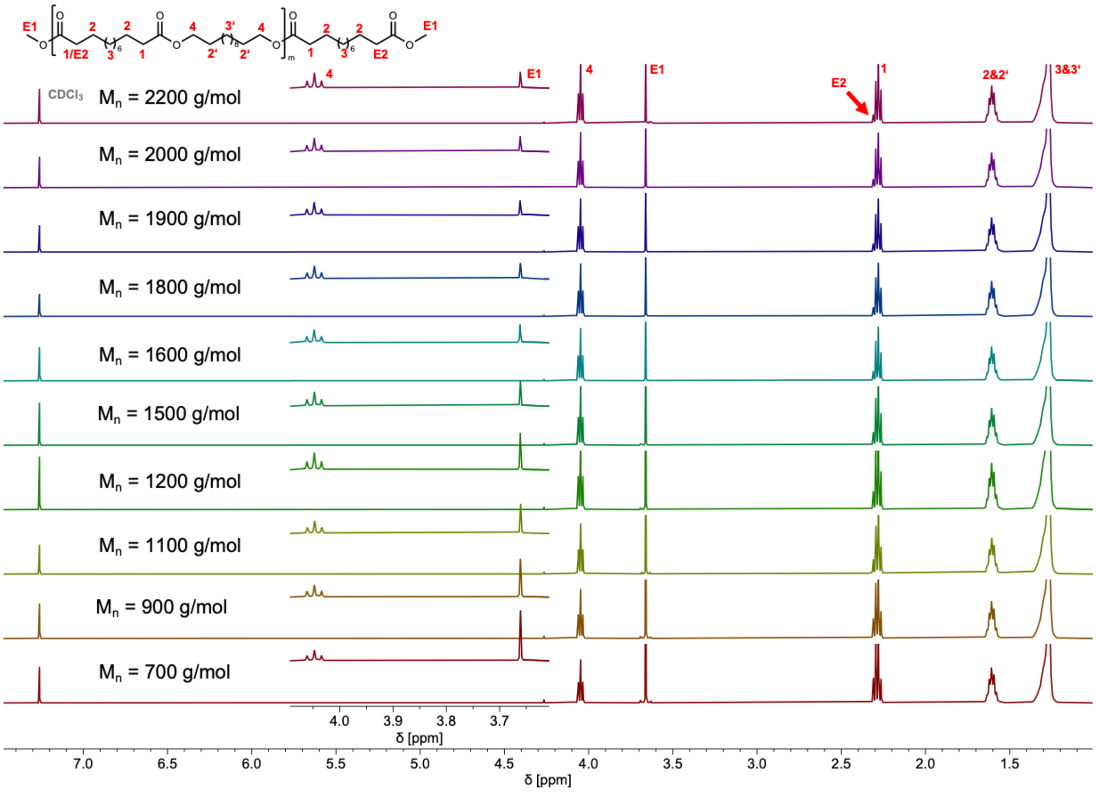

Figure S 3. Stacked ^{1}H NMR spectra (400 MHz, $CDCl_3$, 298 K) of WLE-12,12 waxes with methyl ester end groups. Number-average molar masses M_n were determined *via* end group analysis according to **Equation S 5**.

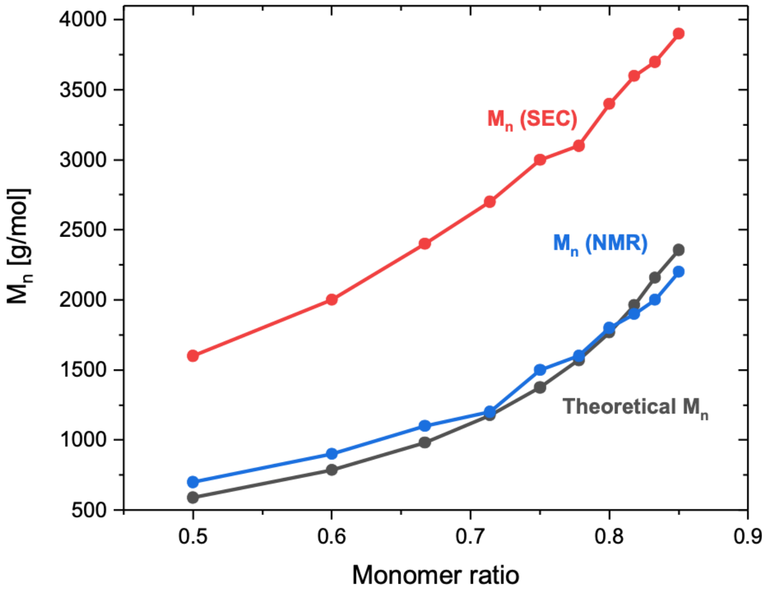

Figure S 4. Relationship between the ratio of C_{12}-diol and C_{12}-dimethyl ester employed in the oligomerization reactions and the theoretically expected molar mass M_n (grey), the molar mass M_n determined by ^{1}H NMR end group analysis (blue), and the molar mass M_n determined by SEC *vs.* PS standards (red).

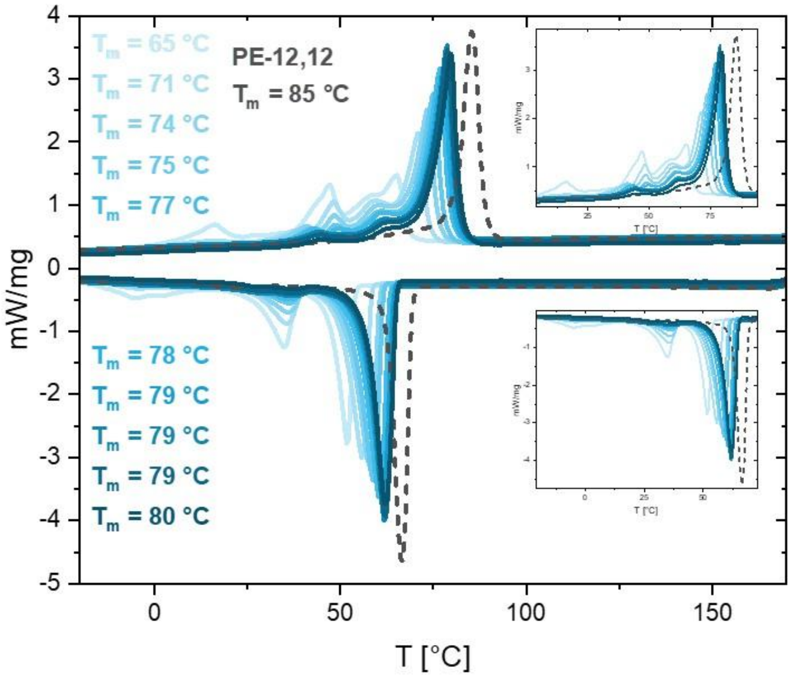

Figure S 5. DSC traces and peak melting temperatures of WLE-12,12 waxes with methyl ester end groups in comparison to reference PE-12,12 (dashed line).

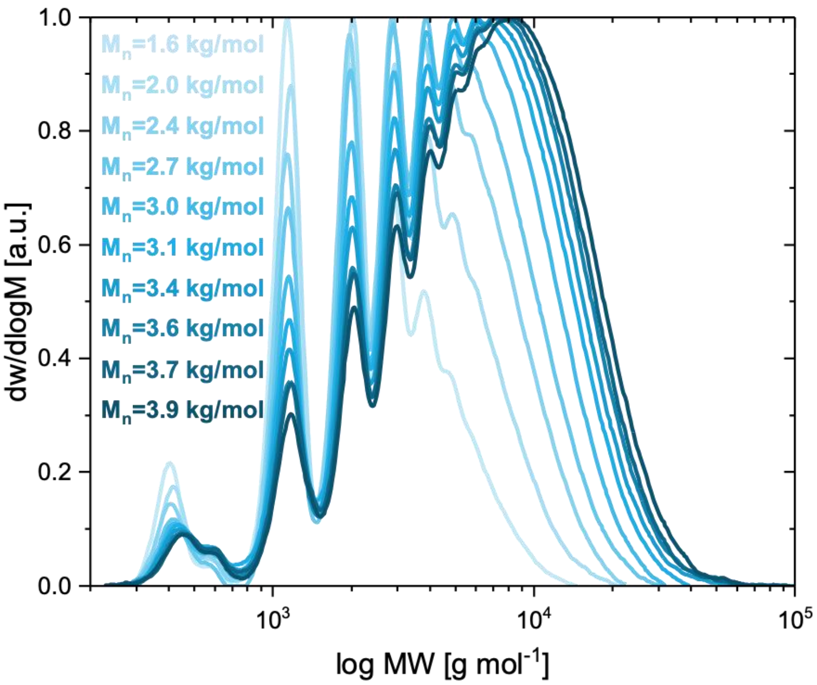

Figure S 6. SEC traces and number-average molar masses M_n *vs.* PS standards of WLE-12,12 waxes with methyl ester end groups (measured in $CHCl_3$ at 35 °C).

Additional characterization data for WLE-12,12 waxes with hydroxy end groups

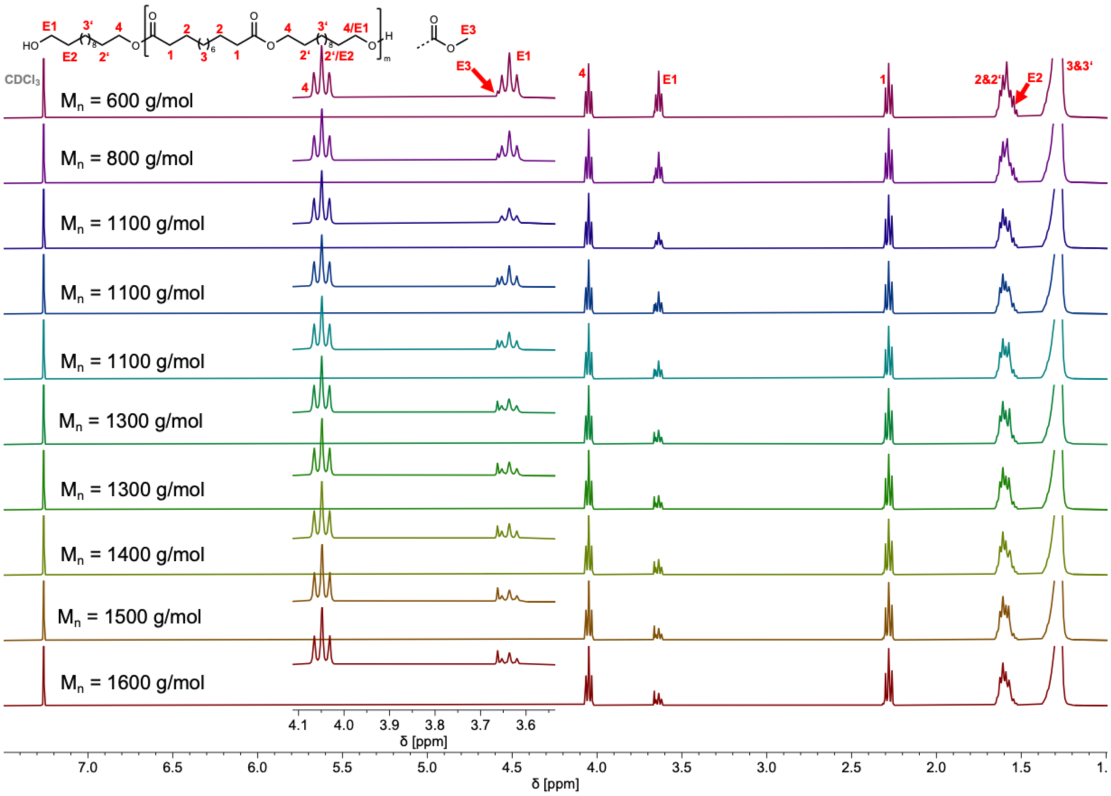

Figure S 7. Stacked ^{1}H NMR spectra (400 MHz, $CDCl_3$, 298 K) of WLE-12,12 waxes with hydroxy end groups. Number-average molar masses M_n were determined *via* end group analysis according to **Equation S 5**.

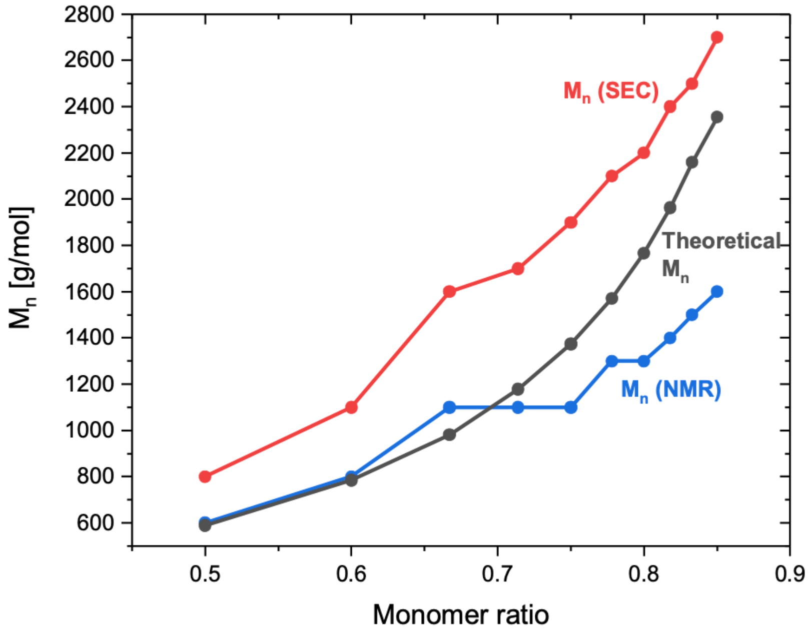

Figure S 8. Relationship between the ratio of C_{12}-dimethyl ester and C_{12}-diol employed in the oligomerization reactions and the theoretically expected molar mass M_n (grey), the molar mass M_n determined by ^{1}H NMR end group analysis (blue), and the molar mass M_n determined by SEC *vs.* PS standards (red).

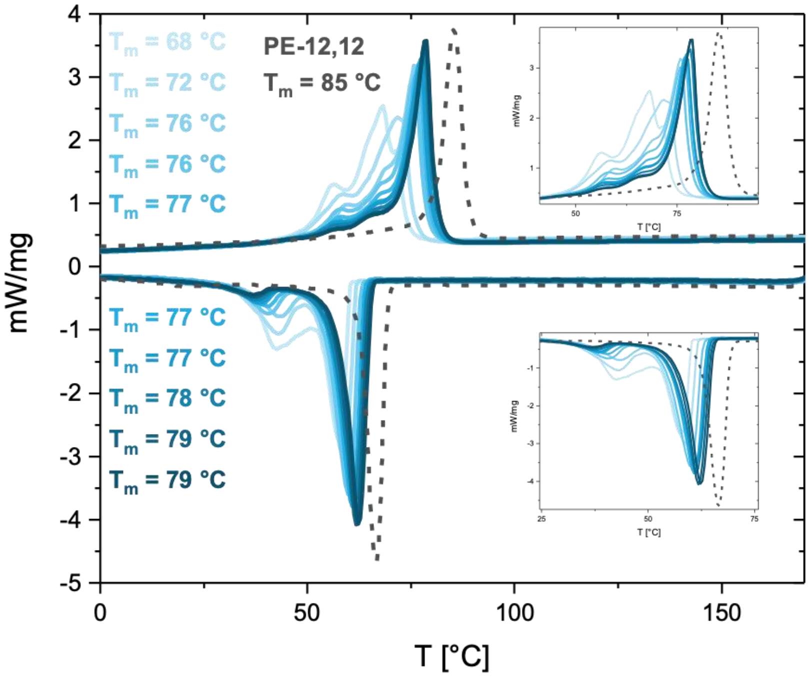

Figure S 9. DSC traces and peak melting temperatures of WLE-12,12 waxes with hydroxy end groups in comparison to reference PE-12,12 (dashed line).

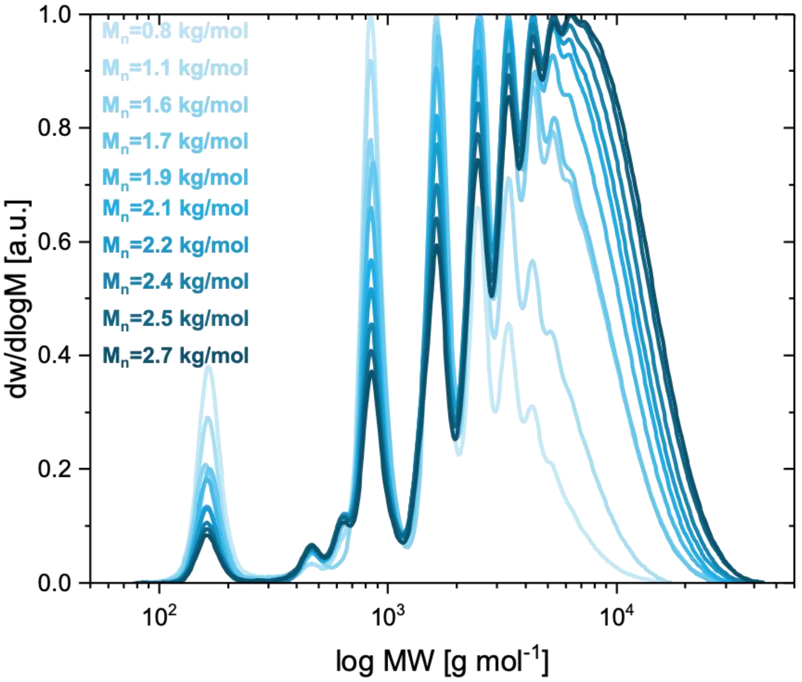

Figure S 10. SEC traces and number-average molar masses M_n *vs.* PS standards of WLE-12,12 waxes with hydroxy end groups (measured in $CHCl_3$ at 35 °C).

Additional characterization data for WLE-12,12 wax synthesized on a larger scale

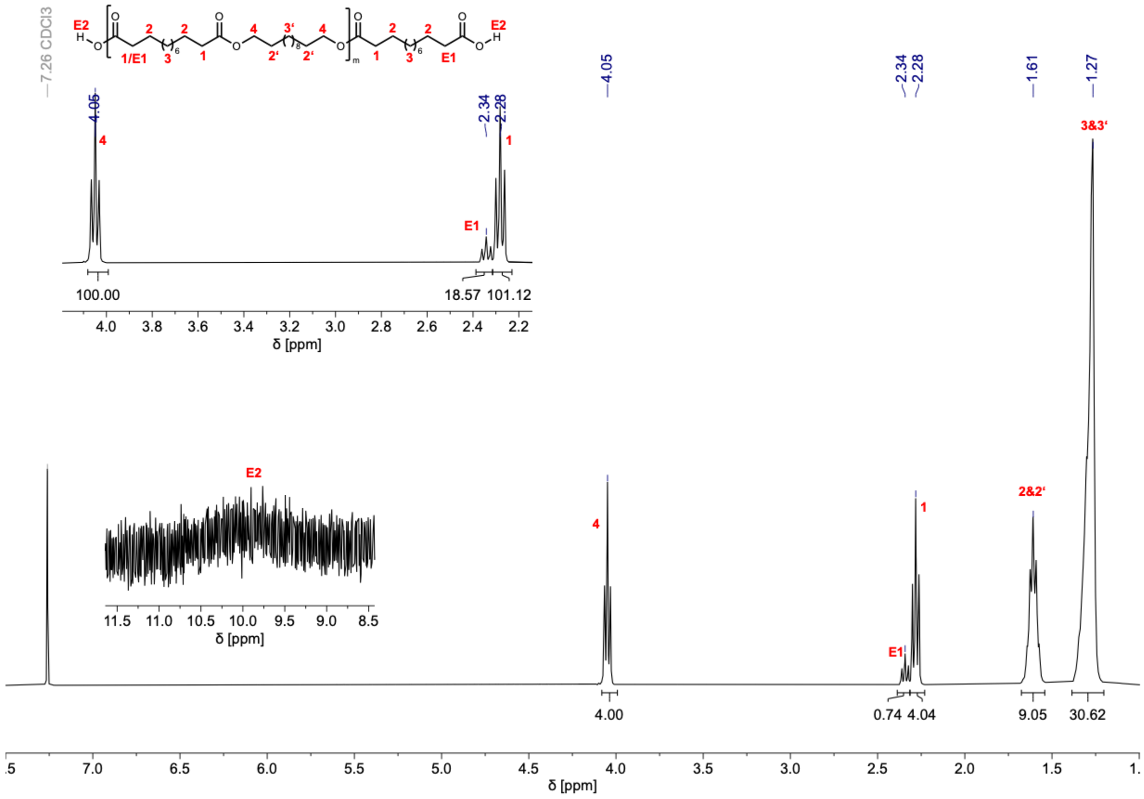

Figure S 11. ^{1}H NMR spectrum (400 MHz, $CDCl_3$, 298 K) of WLE-12,12 wax synthesized on a larger scale. A number-average molar mass of $M_n \approx 2300$ g/mol was determined *via* end group analysis according to **Equation S 5**.

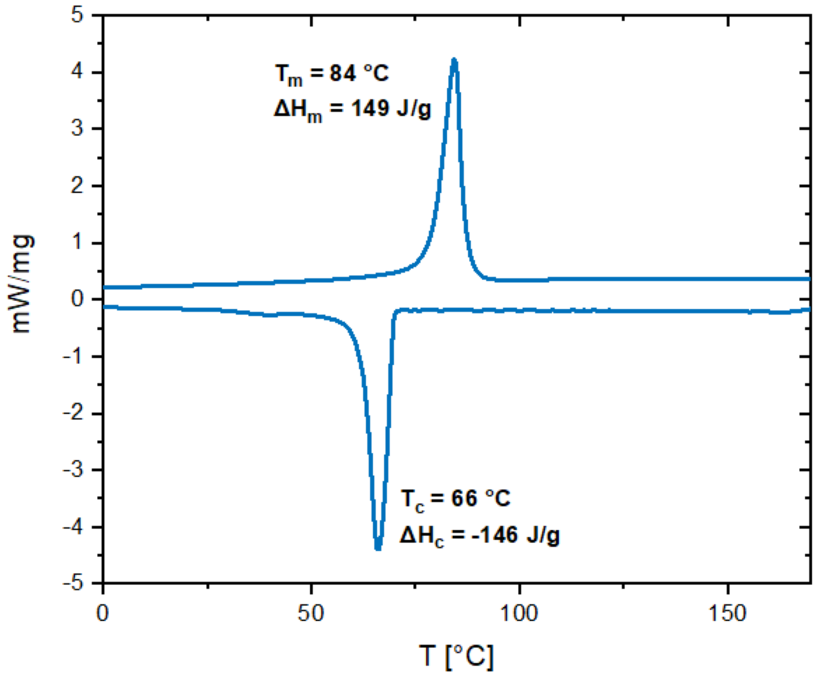

Figure S 12. DSC trace of WLE-12,12 wax synthesized on a larger scale.

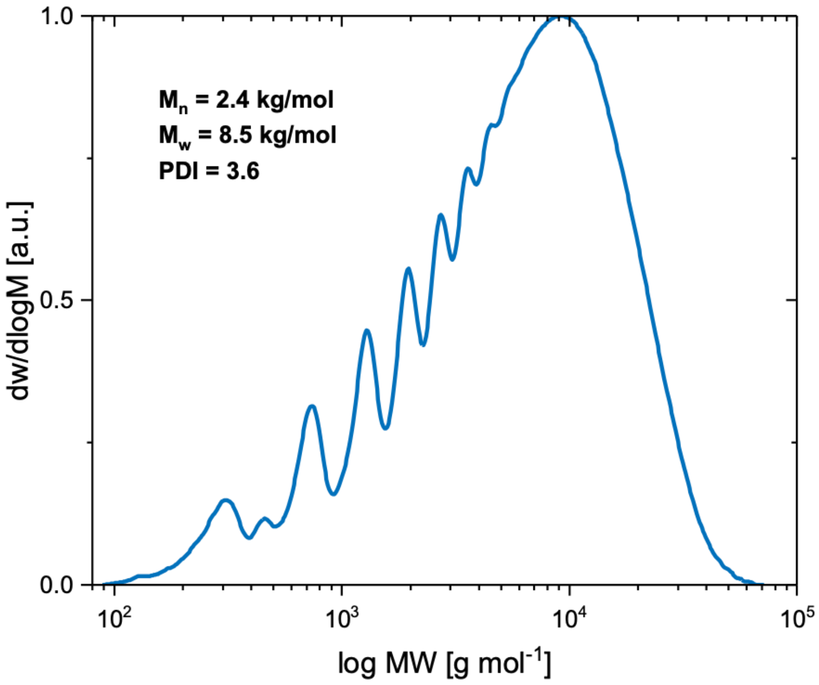

Figure S 13. SEC trace and molar mass data *vs.* PS standards of WLE-12,12 wax synthesized on a larger scale (measured in $CHCl_3$ at 35 °C).

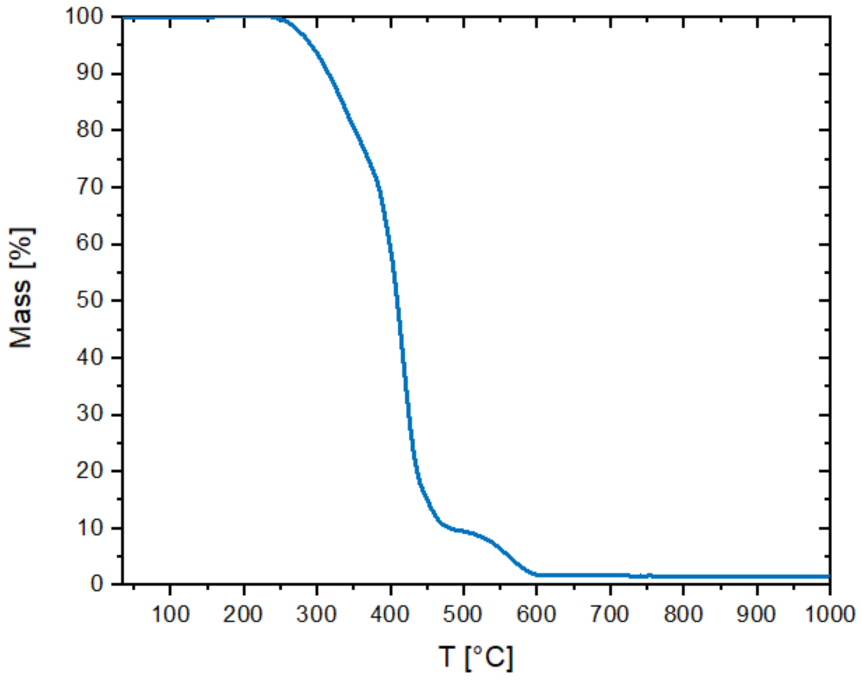

Figure S 14. TGA trace of WLE-12,12 wax synthesized on a larger scale. 5 % weight loss was observed at T ≈ 292 °C.

Table S 1. Contact angles of water and diiodomethane (DIM) and surface free energies of WLE-12,12 with carboxylic acid end groups synthesized on a larger scale.

	Water Contact Angle [°]	DIM Contact Angle [°]	Total Surface Free Energy [mN/m]	Dispersive Surface Free Energy [mN/m]	Polar Surface Free Energy [mN/m]
**WLE-12,12**	82.0 ± 2.5	40.9 ± 1.8	42.1 ± 1.7	39.2 ± 0.9	2.9 ± 0.8

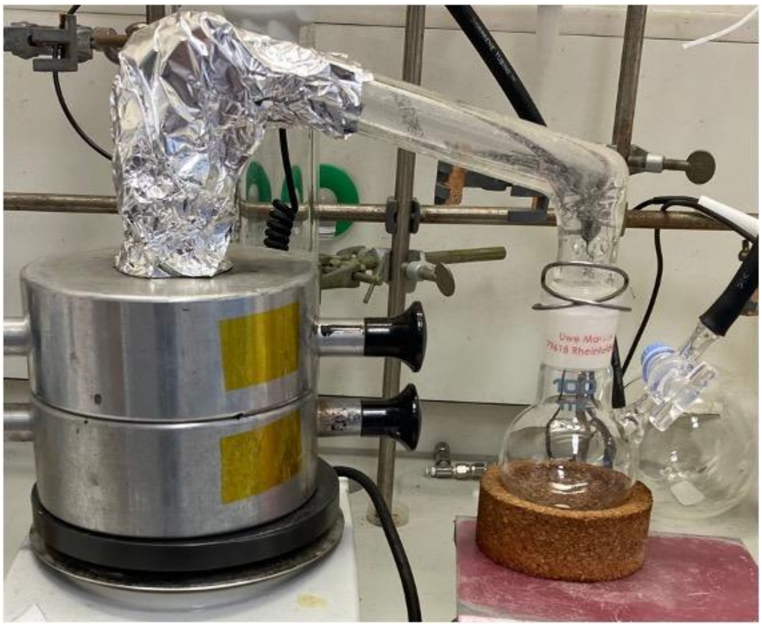

Figure S 15. Experimental setup employed in the synthesis of WLE-12,12 on a larger scale.

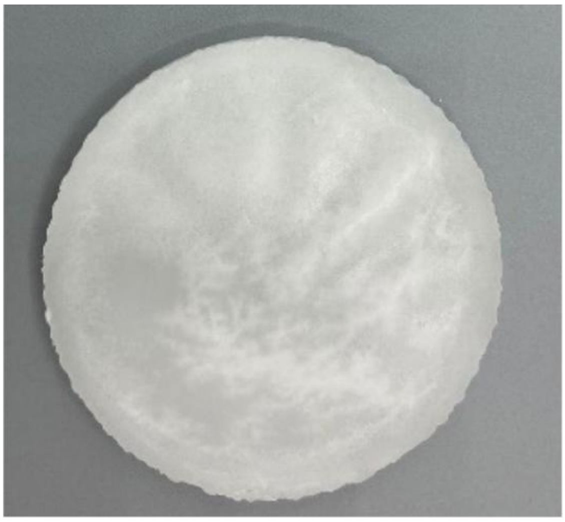

Figure S 16. Photograph of a disc-shaped specimen (diameter ca. 5.5 cm) for WAXS and tensiometry measurements illustrating brittle and colorless WLE-12,12 wax.

Additional characterization data for WLE-2,18 waxes synthesized *via* chain scission

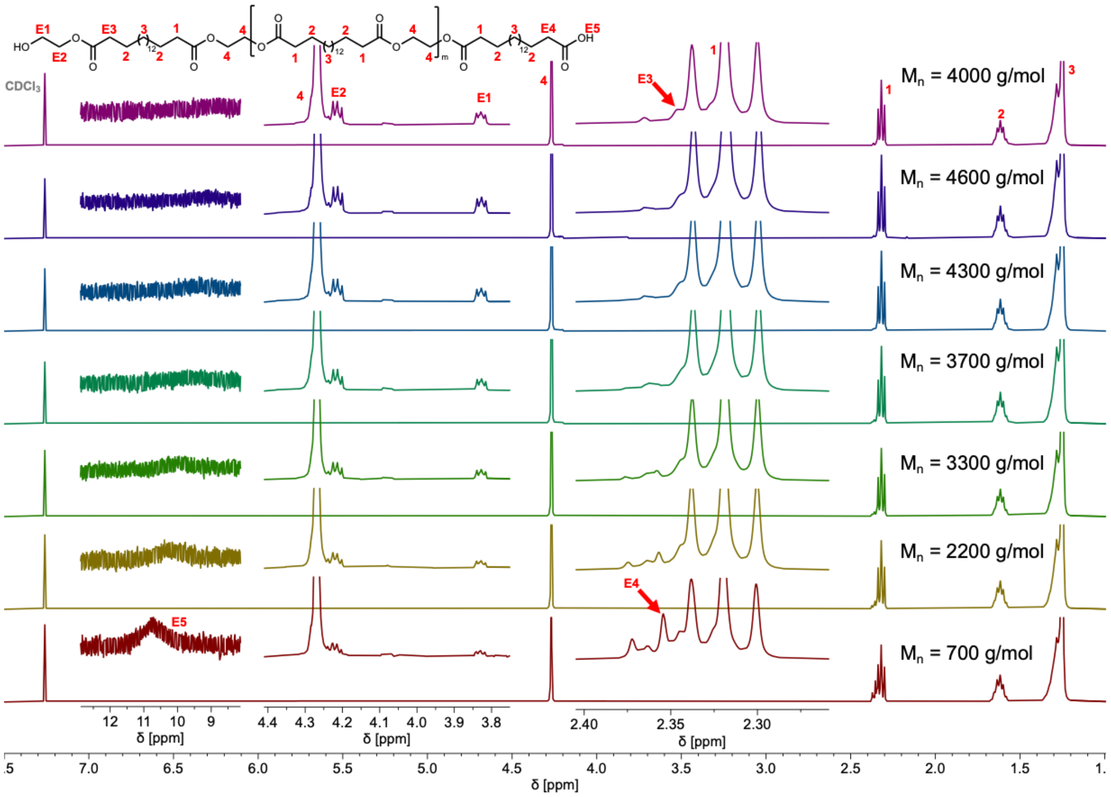

Figure S 17. Stacked ^{1}H NMR spectra (400 MHz, $CDCl_3$, 298 K) of WLE-2,18 waxes synthesized *via* chain scission. Number-average molar masses M_n were determined *via* end group analysis according to **Equation S 7**. Note that the partial overlap of the end group resonances E3 and E4 with resonance 1 results in increasing errors of the determined M_n for higher molar masses.

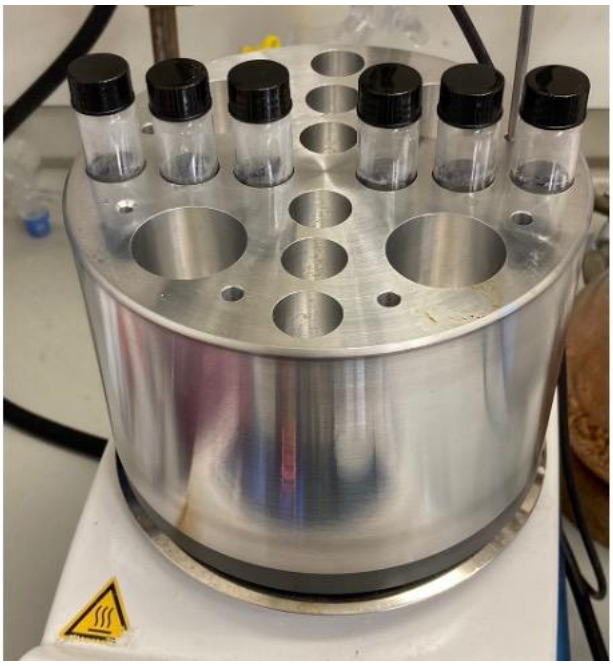

Figure S 18. Experimental setup used for the synthesis of WLE-2,18 waxes on a small scale comprising a heating block and sealed 8 mL glass vials.

Additional characterization data for WLE-2,18 wax synthesized on a larger scale

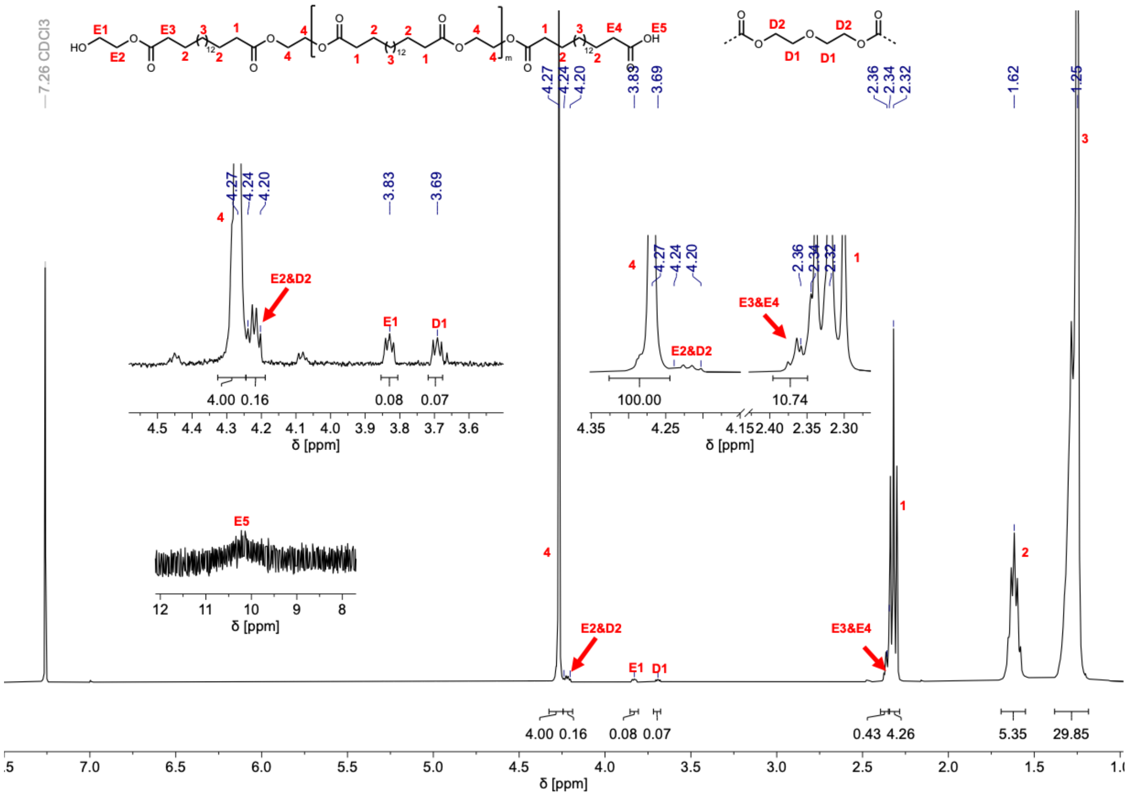

Figure S 19. ^{1}H NMR spectrum (400 MHz, $CDCl_3$, 298 K) of WLE-2,18 wax synthesized on a larger scale. A number-average molar mass of $M_n \approx 1800$ g/mol was determined *via* end group analysis according to **Equation S 7**. Note that small amounts of diethylene glycol units present in the WLE-2,18 wax stem from the synthesis of the PE-2,18 employed.

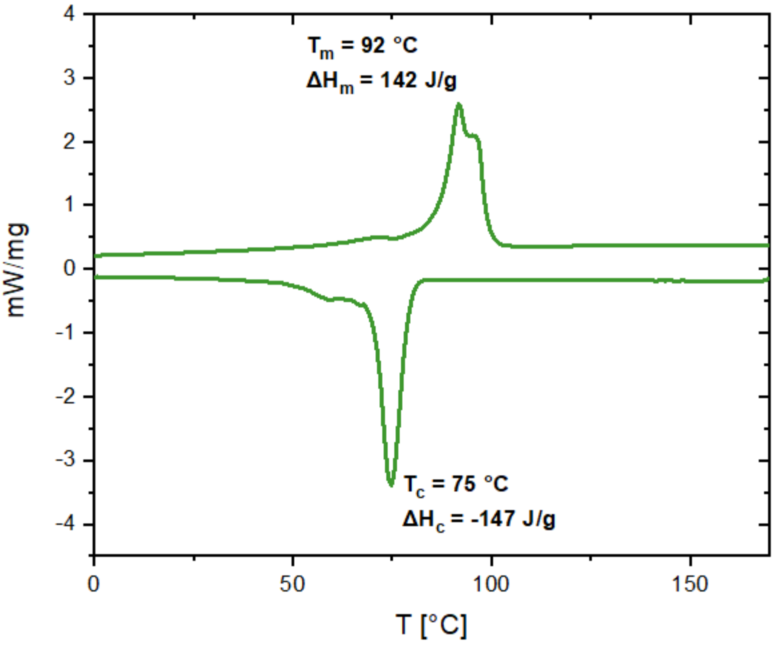

Figure S 20. DSC trace of WLE-2,18 wax synthesized on a larger scale.

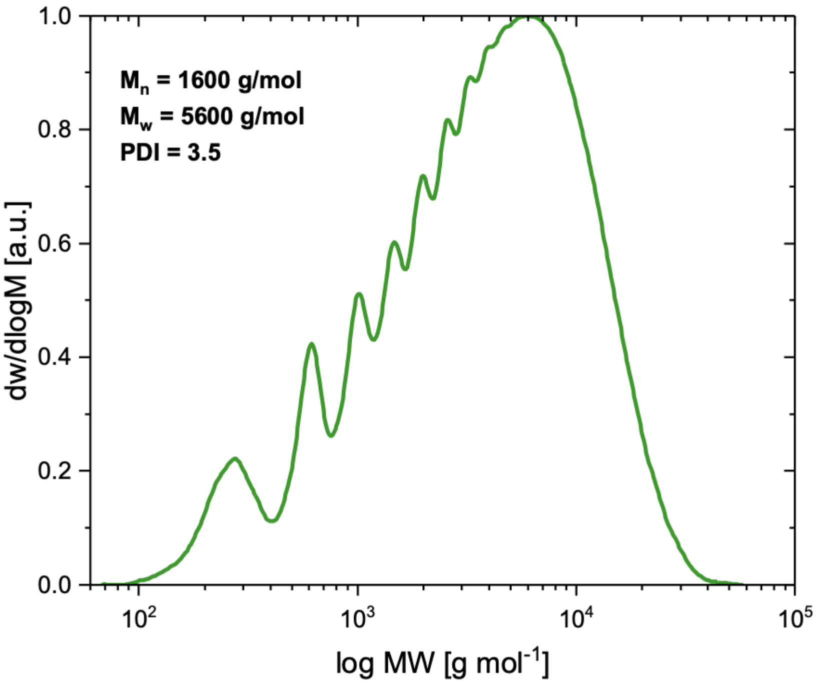

Figure S 21. SEC trace and molar mass data *vs.* PS standards of WLE-2,18 wax synthesized on a larger scale (measured in $CHCl_3$ at 35 °C).

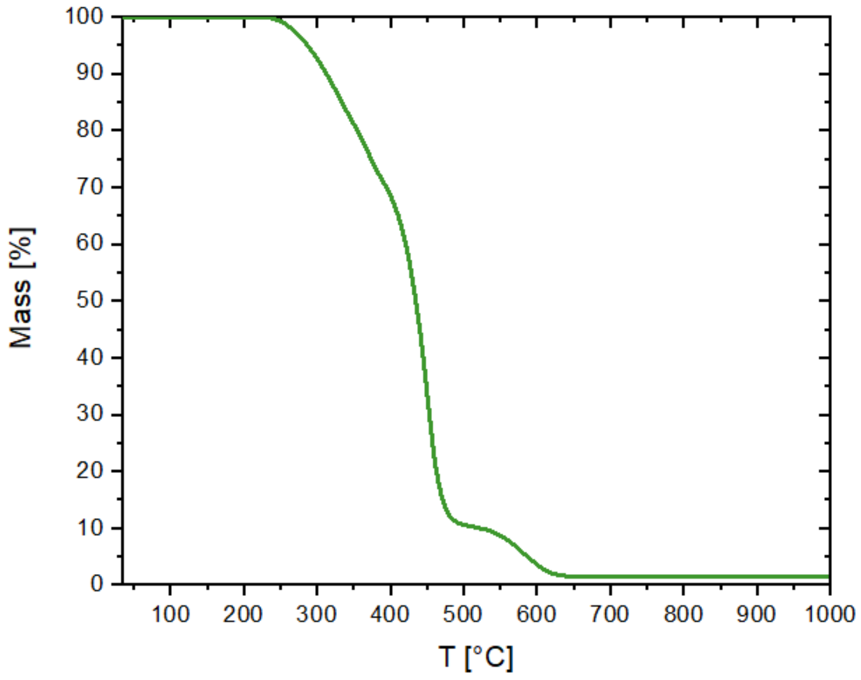

Figure S 22. TGA trace of WLE-2,18 wax synthesized on a larger scale. 5 % weight loss was observed at T ≈ 285 °C.

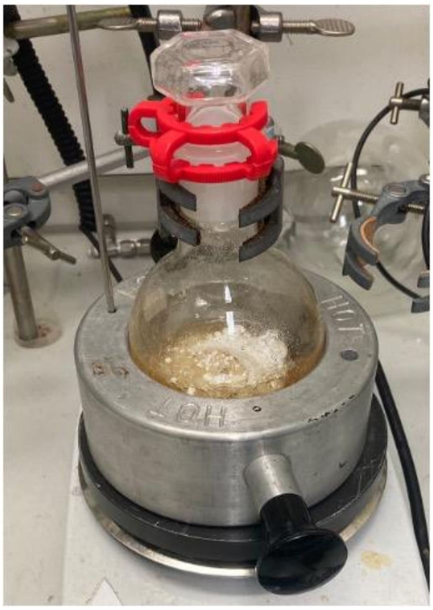

Figure S 23. Experimental setup used for the larger scale synthesis of WLE-2,18 *via* chain scission.

Table S 2. Contact angles of water and diiodomethane (DIM) and surface free energies of WLE-2,18 synthesized *via* chain scission on a larger scale.

	Water Contact Angle [°]	DIM Contact Angle [°]	Total Surface Free Energy [mN/m]	Dispersive Surface Free Energy [mN/m]	Polar Surface Free Energy [mN/m]
**WLE-2,18**	81.4 ± 1.6	39.8 ± 1.0	42.7 ± 1.0	39.7 ± 0.5	3.0 ± 0.5

Determination of molar masses M_n *via* ^{1}H NMR end group analysis

The degrees of polymerization DP_n and molar masses $M_{n,NMR}$ of the synthesized waxes were determined *via* end group analysis of the ^{1}H NMR spectra acquired in deuterated chloroform (400 MHz, 298 K). For the calculation, the integrals of the end group resonances and the integral of a backbone resonance were used.

In general, for the WLE-12,12 waxes the following end group and backbone resonances were employed (cf. **Figure S 1**, **Figure S 3**, **Figure S 7**, **Figure S 11**):

- Carboxylic acid end group (δ = 2.34 ppm, t, -C**H₂**-C(O)-OH): G_1
- Hydroxy end group (δ = 3.64 ppm, t, -C**H₂**-OH): G_2
- Methyl ester end group (δ = 3.66 ppm, s, -CH_2-C(O)-O-C**H₃**): G_3
- Backbone resonance (δ = 4.05 ppm, t, -CH_2-C(O)-O-C**H₂**-): B

The degrees of polymerization DP_n of the WLE-12,12 waxes were determined using **Equation S 4**:

Equation S 4.
$$DP_n = \frac{\int B}{\frac{1}{2}\int G_1 + \frac{1}{2}\int G_2 + \frac{1}{3}\int G_3} + 1$$

On basis of the DP_n, the molar masses $M_{n,NMR}$ were calculated according to **Equation S 5** (cf. **Table S 7**, **Table S 8**, **Table S 9**). The molar mass of the repeat unit of the PE-12,12 waxes $M_{RU\text{-}12,12}$ equals 396.6 g/mol.

Equation S 5.
$$M_{n,NMR} = DP_n * \frac{M_{RU-12,12}}{2}\, g/mol$$

For the WLE-2,18 waxes synthesized by chain scission the following end group and backbone resonances were employed:

- Carboxylic acid end group (δ = 2.36 ppm, t, -C**H₂**-C(O)-OH): G_1
- Hydroxy end group (δ = 2.34 ppm, t, -C**H₂**-C(O)-O-CH_2-CH_2-OH): G_2
- Backbone resonance (δ = 4.27 ppm, s, -CH_2-C(O)-O-C**H₂**-): B

The two end group resonances G1 and G2 overlapped with each other and additionally partially overlapped with the resonance of a backbone methylene group (cf. **Figure S 17**). Therefore, half of the width of the two overlapping end group resonances G1 and G2 was integrated, and the obtained value was multiplied by a factor of two yielding G_{total} (cf. **Figure S 19**). The degrees of polymerization DP_n of the WLE-2,18 waxes were then determined according to **Equation S 6**:

Equation S 6.
$$DP_n = \frac{\int B}{\frac{1}{2}\int G_{total}} + 1$$

On basis of the DP_n, the molar masses $M_{n,NMR}$ were calculated according to **Equation S 7** (cf. **Table S 10**). The molar mass of the repeat unit of the PE-2,18 waxes $M_{RU\text{-}2,18}$ equals 340.5 g/mol.

Equation S 7.
$$M_{n,NMR} = DP_n * \frac{M_{RU-2,18}}{2}\, g/mol$$

Additional characterization data for commercial PE wax

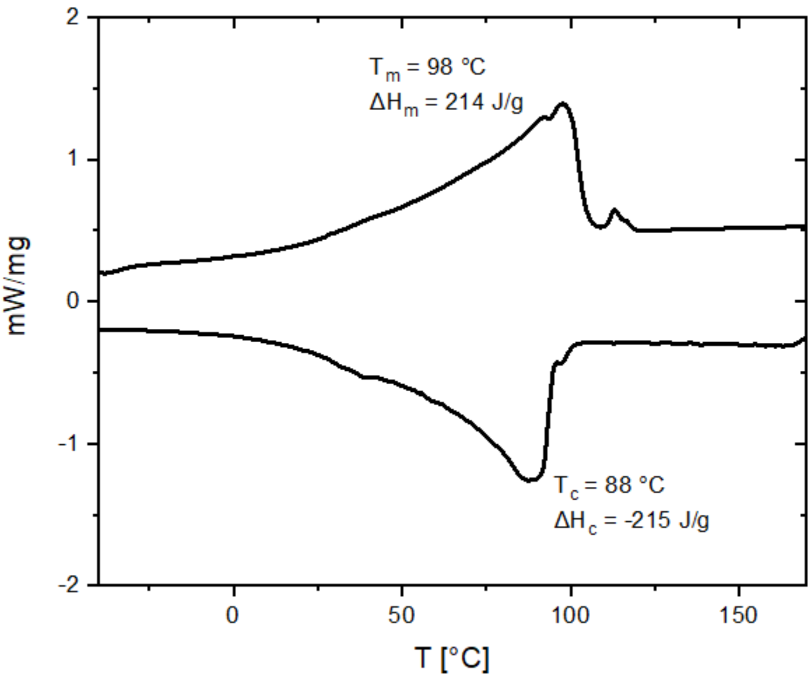

Figure S 24. DSC trace of commercial polyethylene wax (Deurex E 0915 M, strictly linear, with $M_{n,NMR} \approx 700$ g/mol).

S2. Supplementary Tables

Tabular overview of monomer stoichiometries employed in the syntheses of WLE-12,12 waxes

Table S 3. Mass, amount of substance and equivalents (*vs.* 1.0 g (4.3 mmol) C_{12}-diacid) of 1,12-dodecanediol used for the syntheses of PE-12,12 waxes with carboxylic acid end groups and the corresponding theoretically expected DP_n values.

Theoretical DP_n	m C_{12}-diol [mg]	n C_{12}-diol [mmol]	Equivalents
3	439.3	2.2	0.50
4	527.1	2.6	0.60
5	588.6	2.9	0.67
6	623.8	3.1	0.71
7	658.9	3.3	0.75
8	685.3	3.4	0.78
9	702.9	3.5	0.80
10	720.4	3.6	0.82
11	729.2	3.6	0.83
12	746.8	3.7	0.85

Table S 4. Mass, amount of substance and equivalents (*vs.* 1.0 g (3.9 mmol) C_{12}-dimethyl ester) of 1,12-dodecanediol used for the syntheses of PE-12,12 waxes with methyl ester end groups and the corresponding theoretically expected DP_n values.

Theoretical DP_n	m C_{12}-diol [mg]	n C_{12}-diol [mmol]	Equivalents
3	391.6	1.9	0.50
4	469.9	2.3	0.60
5	524.7	2.6	0.67
6	556.1	2.8	0.71
7	587.4	2.9	0.75
8	610.9	3.0	0.78
9	626.5	3.1	0.80
10	642.2	3.2	0.82
11	650.0	3.2	0.83
12	665.7	3.3	0.85

Table S 5. Mass, amount of substance and equivalents (*vs.* 1.0 g (4.9 mmol) C_{12}-diol) of dimethyl 1,12-dodecanedioate used for the syntheses of PE-12,12 waxes with hydroxy end groups and the corresponding theoretically expected DP_n values.

Theoretical DP_n	m C_{12}-diester [mg]	n C_{12}-diester [mmol]	Equivalents
3	638.4	2.5	0.50
4	766.1	3.0	0.60
5	855.5	3.3	0.67
6	906.6	3.5	0.71
7	957.7	3.7	0.75
8	996.0	3.8	0.78
9	1021.0	4.0	0.80
10	1044.2	4.0	0.82
11	1063.6	4.1	0.83
12	1079.9	4.2	0.85

Tabular overview of amount of C_{18}-diacid employed in the syntheses of WLE-2,18 waxes *via* chain scission

Table S 6. Mass and amount of substance of C_{18}-diacid used for the chain scission of 1.0 g PE-2,18 (M_n = 48 kg/mol (SEC *vs*. PS)) to obtain given theoretically expected molar masses M_n.

Theoretical M_n [g/mol]	m C_{18}-diacid [mg]	n C_{18}-diacid [mmol]
1000	458.7	1.46
2000	186.6	0.59
3000	117.1	0.37
4000	85.3	0.27
6000	55.3	0.18
8000	40.9	0.13
10000	32.5	0.10

Tabular overview of thermal and molar mass properties of WLE-12,12 waxes

Table S 7. Overview of thermal and molar mass properties of WLE-12,12 waxes with carboxylic acid end groups. SEC and ^{1}H NMR molar mass data was determined *vs.* PS standards and by end group analysis, respectively. Peak melting temperatures T_m are given.

Theoretical DP_n	Theoretical M_n [g/mol]	M_n (SEC) [g/mol]	M_w (SEC) [g/mol]	PDI	∫ hydroxy	∫ carboxy	DP_n (NMR)	M_n (NMR) [g/mol]	T_m [°C]	ΔH_m [J/g]	T_c [°C]	ΔH_c [J/g]
3	595	500	1100	2.3	0.00	99.09	3.0	600	80	159	65	158
4	793	700	1900	2.7	0.00	69.10	3.9	800	80	158	63	159
5	992	800	2500	3.0	0.00	51.99	4.9	1000	81	159	64	160
6	1190	1000	3200	3.2	0.00	42.45	5.7	1100	82	155	64	156
7	1388	1100	3800	3.4	1.08	36.06	6.4	1300	83	153	64	155
8	1586	1300	4600	3.6	1.19	30.69	7.3	1400	83	151	65	151
9	1785	1600	5300	3.4	1.18	26.47	8.6	1700	83	148	65	149
10	1983	1700	6000	3.5	1.41	25.48	8.4	1700	83	154	66	154
11	2181	1700	6400	3.8	1.60	23.48	9.0	1800	83	148	65	149
12	2380	1800	6800	3.9	1.69	21.73	9.5	1900	83	149	65	150
12*	2380	2400	8500	3.6	0.00	18.57	11.8	2300	84	149	66	146

* WLE-12,12 synthesized on a larger scale.

Table S 8. Overview of thermal and molar mass properties of WLE-12,12 waxes with methyl ester end groups. SEC and ^{1}H NMR molar mass data was determined *vs.* PS standards and by end group analysis, respectively. Peak melting temperatures T_m are given.

Theoretical DP_n	Theoretical M_n [g/mol]	M_n (SEC) [g/mol]	M_w (SEC) [g/mol]	PDI	∫ hydroxy	∫ ester	DP_n (NMR)	M_n (NMR) [g/mol]	T_m [°C]	ΔH_m [J/g]	T_c [°C]	ΔH_c [J/g]
3	595	1600	2700	1.7	-	127.67	3.4	700	65	154	52	156
4	793	2000	3600	1.8	-	87.78	4.4	900	71	159	56	158
5	992	2400	4400	1.8	-	64.57	5.4	1100	74	158	58	159
6	1190	2700	5100	1.9	-	57.48	6.2	1200	75	158	59	158
7	1388	3000	5800	2.0	-	47.20	7.4	1500	77	158	61	158
8	1586	3100	6500	2.1	-	41.01	8.3	1600	78	158	61	157
9	1785	3400	7100	2.1	-	37.80	8.9	1800	79	154	62	155
10	1983	3600	7700	2.2	-	34.30	9.8	1900	79	155	62	155
11	2181	3700	7900	2.2	-	33.01	10.1	2000	79	154	62	154
12	2380	3900	8500	2.2	-	30.25	10.9	2200	80	146	62	145

Table S 9. Overview of thermal and molar mass properties of WLE-12,12 waxes with hydroxy end groups. SEC and ^{1}H NMR molar mass data was determined *vs.* PS standards and by end group analysis, respectively. Peak melting temperatures T_m are given.

Theoretical DP_n	Theoretical M_n [g/mol]	M_n (SEC) [g/mol]	M_w (SEC) [g/mol]	PDI	∫ hydroxy	∫ ester	DP_n (NMR)	M_n (NMR) [g/mol]	T_m [°C]	ΔH_m [J/g]	T_c [°C]	ΔH_c [J/g]
3	595	800	2100	2.7	97.10	5.54	3.0	600	68	162	58	161
4	793	1100	2900	2.6	67.82	4.80	3.8	800	72	160	59	161
5	992	1600	4300	2.7	44.61	1.58	5.4	1100	76	161	61	164
6	1190	1700	4300	2.6	42.62	5.82	5.3	1100	76	157	60	160
7	1388	1900	4700	2.6	38.52	6.32	5.7	1100	77	155	61	160
8	1586	2100	5200	2.4	30.97	6.77	6.6	1300	77	155	61	157
9	1785	2200	5400	2.5	30.22	7.53	6.7	1300	77	152	61	156
10	1983	2400	5800	2.4	27.94	7.71	7.1	1400	78	152	61	155
11	2181	2500	6300	2.5	24.48	8.05	7.7	1500	79	152	62	157
12	2380	2700	6600	2.5	23.77	7.56	7.9	1600	79	157	62	156

Tabular overview of thermal and molar mass properties of WLE-2,18 waxes

Table S 10. Overview of thermal and molar mass properties of WLE-2,18 waxes synthesized *via* chain scission. SEC and ^{1}H NMR molar mass data was determined *vs.* PS standards and by end group analysis, respectively. Peak melting temperatures T_m are given.

Theoretical M_n [g/mol]	M_n (SEC) [g/mol]	M_w (SEC) [g/mol]	PDI	∫ end groups*	DP_n (NMR)	M_n (NMR) [g/mol]	T_m [°C]	ΔH_m [J/g]	T_c [°C]	ΔH_c [J/g]
1000	1100	4400	4.1	67.40	4.0	700	90	143	73	148
2000	2400	9200	3.8	16.54	13.1	2200	92	135	73	140
3000	3000	12100	4.1	10.98	19.2	3300	93	135	76	143
4000	4100	15500	3.8	9.62	21.8	3700	93	130	75	137
6000	7000	19200	2.7	8.26	24.2	4300	93	123	75	132
8000	8400	17800	2.1	7.66	27.1	4600	94	128	76	133
10000	9800	21700	2.2	9.00	23.2	4000	95	129	76	135
2000**	1600	5600	3.5	21.48	10.3	1800	92	142	75	147

* The resonances were integrated by half and the obtained values were multiplied by a factor of two. The multiplied values are given.
** WLE-2,18 synthesized on a larger scale.

References

(1) Flory, P. J. Fundamental principles of condensation polymerization. *Chem. Rev.* **1946**, *39* (1), 137–197. DOI: 10.1021/cr60122a003.

(2) Geyer, B.; Röhner, S.; Lorenz, G.; Kandelbauer, A. Designing oligomeric ethylene terephtalate building blocks by chemical recycling of polyethylene terephtalate. *J. Appl. Polym. Sci.* **2014**, *131* (2), 39786. DOI: 10.1002/app.39786.
